# Knee Biomechanics During Neurocognitively Challenged Drop Landings in Male Elite Soccer Players with Anterior Cruciate Ligament Reconstruction

**DOI:** 10.1186/s40798-024-00685-w

**Published:** 2024-02-27

**Authors:** Ghazal Mohammad Gholipour Aghdam, Mohammad Hossein Alizadeh, Hooman Minoonejad, Elham Shirzad, Jan Wilke

**Affiliations:** 1 Department of Sports Injury and Biomechanics, Faculty of Sport Sciences and Health, University of Tehran, Tehran, Iran; 2 Department of Movement Sciences, University of Klagenfurt, Universitätsstraße 65-67, 9020 Klagenfurt, Austria

**Keywords:** Neurocognition, Injury, Decision making, Anterior cruciate ligament reconstruction, Athletes

## Abstract

**Background:**

Reactive decision-making during athletic movement has been demonstrated to evoke unfavorable biomechanics associated with anterior cruciate ligament (ACL) rupture. However, the current evidence is based on assessments of healthy individuals. We aimed to investigate unplanned jump landing kinetics and knee kinematics in ACL-reconstructed (ACLR) and non-injured athletes.

**Methods:**

A total of 30 male professional soccer players (n = 15 ACLR after return to play, n = 15 matched controls) performed six drop landings onto a force plate. As a neurocognitive challenge requiring decision-making, a diode flashing in randomly selected colors indicated the requested landing location. Knee joint kinematics (flexion, valgus and tibial rotation angles) assessed with a 10-camera motion capture system, vertical ground reaction force (vGRF), time to stabilization (TTS) and length of the center of pressure (COP) trace (all analyzed from force plate data) were calculated. Cognitive function was assessed using the CNS Vital Signs battery.

**Results:**

The ACLR group produced lower knee flexion angles than the control group (median [interquartile range] 50.00° [6.60] vs. 55.20° [4.45], *p* = .02). In addition, path length of the center of pressure (379 mm [56.20] vs. 344 mm [37.00], *p* = .04) and ground reaction force (3.21 N/kg [0.66] vs. 2.87 N/kg [0.48], *p* = .01) were higher for the ACLR group. No differences were found for knee valgus (*p* = .96), tibial rotation (*p* = .83) and TTS (*p* = .82). ACLR participants scored lower for reaction time (*p* = .02) and processing speed (*p* = .01). Unfavorable knee biomechanics were more often related to cognitive function in the ACLR group than in the control group (*p* < .05).

**Conclusions:**

Impaired reactive decision-making during athletic movement may contribute to the high re-injury risk in individuals with ACLR. Prospective studies confirming potential cause-effect relationships are warranted.

## Highlights

- ACL-reconstructed (ACLR) athletes display impaired biomechanics in reactive drop landings.
- Decreased landing safety is linked to deficits in cognitive function.
- The development of specific training measures may help to reduce injury risk.

## Background

Ruptures of the anterior cruciate ligament (ACL) count among the most devastating injuries in open-skill sports [1]. The vast majority of the torn ligaments are surgically reconstructed and an estimated 81% of the athletes return to sports. However, it has been shown that only one in two individuals, irrespective of pre-injury levels, manage to resume competitive play [2]. In addition to the high probability of an unsuccessful comeback, the risk of a second ACL tear may be as high as 23% in young athletes [3]. Both observations are worrisome considering the high efforts spent during the rehabilitation process.

Traditionally, diagnostic testing and training in the return-to-play period have focused on motor abilities such as strength, power, range of motion, and coordination [4]. However, there is accumulating evidence that the consequences of ACL rupture reach beyond these capacities. For instance, previous research revealed a peripheral de-afferentation induced by the physical destruction of proprioceptors located in the ACL and the knee joint capsule [5]. The resulting changes in kinesthetic input lead to modified cortical activation patterns [6] which, in the end, could affect motor control. This is of importance because the described deficiencies may not (1) be resolved using classical exercise approaches and (2) be detected by classical testing paradigms [7].

Athletes in team sports act in a highly complex environment, requiring rapid adaptations to constantly changing situational demands. The ability to prevent an injury has therefore been suggested to depend substantially on neurocognitive function [4, 7]. Indeed, a recent systematic review showed that reaction time is a predictor of lower limb injury [4]. However, neither isolated motor performance assessments nor most functional tests (e.g. Landing Error Scoring System [8] or Functional Movement Screen [8]) include significant reactive components. In view of the accumulating evidence suggesting a prominent role of neurocognitive function in injury risk, several studies have proposed motor assessments requiring spontaneous decision-making [9]. When compared to pre-planned athletic movements (e.g. cuts, changes of directions) without a reactive component, identical actions with spontaneous decision-making induced changes in knee biomechanics which are associated with ACL injury (i.e., decreased knee flexion and increased valgus moments [9]). However, most available trials focused on healthy participants. To the best of our knowledge, only Giesche et al. [10] examined athletic movements with decision-making in injured individuals, not reporting a difference in jump-landing mechanics of controls and ACLR athletes. Despite the pioneering work, they assessed only few biomechanical variables such as time to stabilization and vertical ground reaction force but did not capture knee-related outcomes (i.e., joint angles). As a consequence, it is not clear if differences between uninjured athletes and individuals with ACLR are knee-specific. Against this background, this study aimed to compare knee kinematics and kinetics during neurocognitively challenged drop landings in ACLR athletes. We hypothesized (1) that individuals with ACLR, compared to non-injured controls, would exhibit knee biomechanics which are more suggestive of ACL injury (e.g., less flexion/more valgus, higher ground reaction forces) and (2) that these altered biomechanics are related to impairments in neurocognitive function (e.g., memory, processing speed).

## Methods

We performed a matched-pairs trial recruiting a control group of healthy athletes (CON) and a group of individuals with ACLR. Both, CON and ACLR participants performed a series of neurcognitively challenged single-leg drop landings (SDL) onto a force platform.

### Sample

A total of n = 30 male professional soccer players with a mean age of 22.00 ± 1.80 years and a body mass index of 23.00 ± 0.90 kg/m2 (Table 1) were included. We compared two groups: The first (ACLR; n = 15) had successfully returned to competitive play after sustaining a non-contact ACL tear and undergoing surgical reconstruction. The CON group (n = 15) was matched for sex, age (± 2 years), body mass index (± 1 kg/m^2^), and performance level (same league ± 5 played games). For study inclusion, individuals in the ACLR group had to have a Tegner score of seven points or higher and a minimum of two years had to be passed since surgical reconstruction. This time frame was chosen because revision surgery, indicating an unusually complex or unsuccessful rehabilitation process, is frequently performed between the first and second year [3]. All participants (ACLR and CON group) were regular players of the post premier league which represents the highest performance level in Iran. Individuals with a history of lower limb surgery or lower limb injury (except for the ACL injury in ACLR), vestibular dysfunction, impaired vision, pain, presence of delayed onset muscle soreness, sleep problems, and skin disorders preventing the attachment of markers were excluded.

**Table 1:** Participant characteristics

	ACLR	CON	Group difference (t-test)	Effect Size *(Cohen's d)*
Participants (n)	15	15	–	–
Height (cm)	174.80 ± 5.40	176.60 ± 4.00	*p* = 0.52	0.18
Body weight (kg)	70.10 ± 5.10	73.10 ± 6.70	*p* = 0.40	0.32
Body mass index (kg/m^2^)	22.90 ± 0.61	23.40 ± 1.40	*p* = 0.48	0.16
Age (years)	22.30 ± 2.10	23.50 ± 2.30	*p* = 0.39	0.27
Beck anxiety (score)	2.83 ± 2.78	1.83 ± 1.83	*p* = 0.48	0.31
PSQI (score)	2.50 ± 1.87	3.33 ± 2.16	*p* = 0.49	0.28
Time since surgery (months)	42.83 ± 10.06	–	–	–

Table shows mean and standard deviation. ACLR = anterior cruciate ligament reconstruction, CON = control, cm = centimeters, kg = kilograms, m = meters, PSQI = Pittsburgh Sleep Quality Index

### Experimental Task

All participants performed a general (30 jumping jacks) and a specific warm-up (5 single-leg drop landings/ SDL, see below). The actual experiment consisted of six neurocognitively challenged SDL from a 30-cm box onto a force plate. The neurocognitive component was implemented using an LED light positioned at two meters distance in front of the participants. The LED was connected to a custom-made footswitch mat on the box, which was activated as soon the participants’ feet left the box (sensor latency: 20 ms). This fast activation ensured the stimulus delivery during the flight, which had an average duration of 330 ± 3 ms (determined using a slow-motion camera (PowerShot SD 1400, Canon, Tokyo, Japan) with 30 fps and Kinovea 0.8.15 software [11]). The landing area (force plate with 50 × 50 cm) consisted of three equidistant parts, which each had a specific color (red, green, and blue) corresponding to the colors shown by the LED. Participants were instructed to land with the foot aligned into the correct direction. For instance, if the LED lighted up in red, the foot had to be directed towards the red light.

**Fig. 1:**
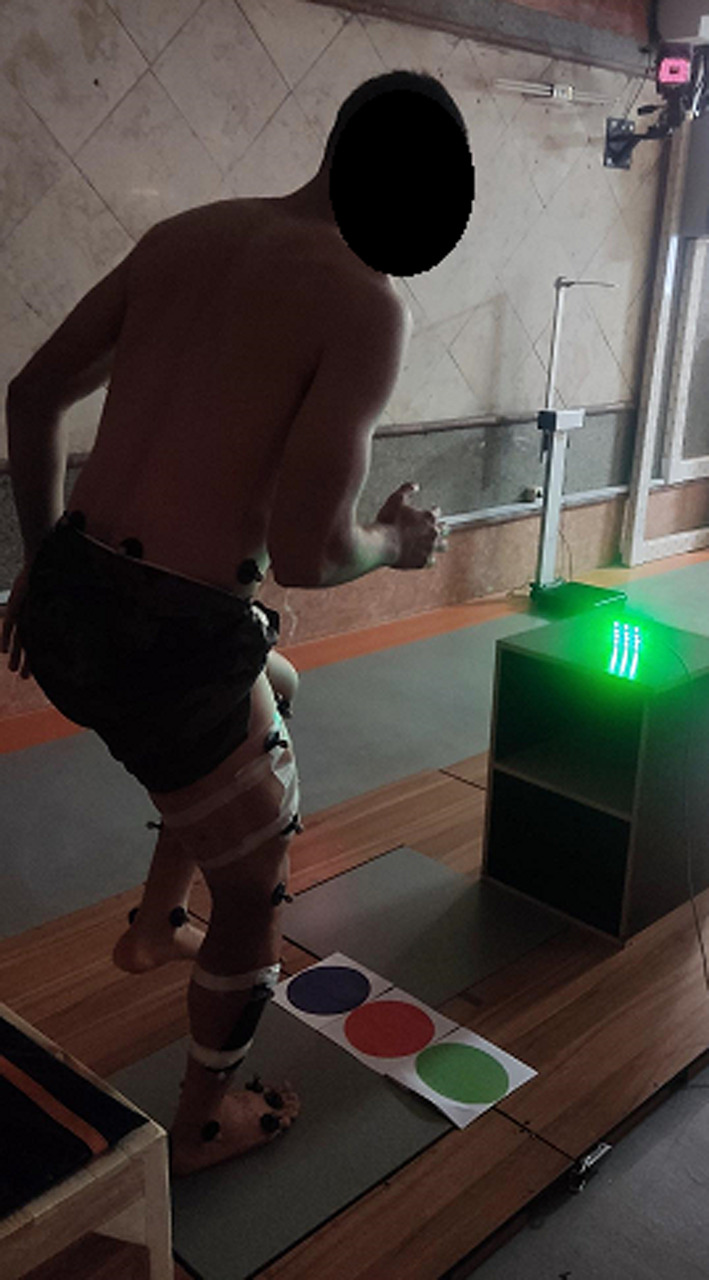
Depiction of the experimental set-up. Participants performed one-legged drop landings from a box with a footswitch mat (bottom left) onto a force plate. The required landing location was indicated (trigger: footswitch mat) by an LED light (lighting green in this example) and colored strips on the ground

When jumping, the athletes had to attentively monitor the light and, after reacting to the LED, to as quickly as possible stabilize the landing position. The arms could be used to equilibrate the postural sway. The view did not have to be fixated on the LED after recognizing its color. The selection of the LED color was randomly chosen for each trial but the order was different for all participants to avoid the possibility of under-prediction about the target color. The number of landings per foot direction (left/right/straight) was the same for all participants. SDL were classified as successful if the participants’ feet were aligned with the correct color, and if they remained stationary for 15 s without touching the ground with the free leg. All individuals performed the SDL’s at their own pace and special care was given not to perform jumps with insufficient rest in-between. Before each jump, participants were asked if they were sufficiently rested. If they were unsure, a 0–10 numerical rating scale (0 = fully rested state) was used as an orientation and jumps were only performed if ratings were 3 or lower. Landings were performed using the non-dominant leg because in soccer, the dominant leg would normally be used for ball handling/kicking. The experimental set-up is displayed in Fig. 1.

### Biomechanical Outcomes

A 50 × 50 cm force plate (Accugait, AMTI Inc., Watertown, USA) with a sampling frequency of 1,000 Hz was used to assess landing kinetics. Three parameters were calculated from raw data according to Fransz et al. [12]. Vertical peak ground reaction force (vGRF), normalized to body weight, was measured as the integral between 0 and 40 ms after the initial foot contact to the force plate [13]. To determine time to stabilization (TTS), a cumulative average of the vGRF, registered over the 15 s after landing, was calculated. TTS was then determined as the point where the cumulative average no longer surpassed the threshold of 0.25 standard deviations of the vGRF’s overall series mean [14]. Finally, center of pressure (COP), corresponding to the total (media-lateral and anterior–posterior) distance [mm] covered by the COP, was calculated within the first 2.5 s after landing, as this duration represents the early landing phase.

We used a camera-based 3D motion capture system (Raptor E cameras, Motion Analysis Corporation, Oxford, United Kingdom) to measure knee joint kinematics. Seventeen infrared reflective markers (14 mm) were attached to the lower leg being measured and the trunk of the participants using a point cluster technique [15]. The coordinates of the markers were recorded from 30 s before to 30 s after the SDL and this time frame was used for kinematic analyses. The reference position for the measurements was obtained during static standing. The moment of the first force registration of the force plate (vGRF > 10 N) was considered the initial contact during landing. Here, maximum angular displacement of the knee (flexion, valgus) and tibial internal rotation during landing (initial contact) were calculated. Ten infrared cameras (Raptor E cameras and Cortex 7 software, both Motion Analysis Corporation, Oxford, United Kingdom) were used for recordings with a sampling frequency set to 200 Hz. The force-plate was synchronized with the camera system. Live marker tracking was performed using digital software algorithms (Cortex 7) in order to extract three-dimensional marker coordinates. Data from each SDL trial were then exported into MATLAB (MathWorks Inc., Natick) for further analysis. Marker coordinates and vertical ground reaction forces (vGRF), assessed with the force plate, were low-pass filtered at a cutoff frequency of six Hz, respectively, using a zero-lag fourth-order Butterworth filter. The Cardan-Euler method was used to calculate the 3-dimensional angles of the hip and knee joints [16]. We applied an X–Y-Z Cardan rotation sequence to calculate joint angles. This sequence involved three steps: first, rotation around the laterally directed axis (X); second, rotation around the anteriorly directed axis (Y); third, rotation around the vertically directed axis (Z).

First, the matrix rotation of the pelvis, thigh, and shank segments were computed [17]. Joint angles were determined as the orientation of the distal segment to the orientation of the proximal segment. The rotation matrix of the joint was calculated by multiplying the distal segment rotation matrix in the transpose of the proximal segment rotation matrix. For instance, the knee rotation matrix was calculated using the following equation:

$${\text{R}}_{{{\text{knee}}}}\;{\text{R}}_{{{\text{shank}}}} {\text{R}}_{{{\text{thigh}}}}{\prime}$$

where R_knee is the knee rotation matrix (3 $\times$ 3 matrix), R_shank]is the shank rotation matrix, and R’_thigh is the transpose of the thigh rotation matrix. The cardan angles were derived from the joint rotation matrix using the following equations:

$$\alpha ={{\text{tan}}}^{-1}\frac{{-R}_{32}}{{R}_{33}}$$

$$\beta ={{\text{tan}}}^{-1}\frac{{R}_{31}}{\sqrt{{R}_{11}^{2}+{R}_{21}^{2}}}$$

$$\gamma ={{\text{tan}}}^{-1}\frac{{-R}_{21}}{{R}_{11}}$$

According to the X–Y-Z rotation sequence, $\alpha$, $\beta$, and $\gamma$ represent flexion–extension, abduction–adduction, and axial rotation, respectively. As indicated, maximum angular displacement of the knee in the sagittal, frontal, and transverse planes during the initial contact with the force plate were calculated and initial contact was defined as the moment when vGRF exceeded 10 N [18].

### Questionnaire Outcomes

The Beck Depression Short Inventory (BDI-S (was used to assess participants' depression and anxiety status. It includes thirteen 4-point-likert scale *questions (0 to 3*). A total score of ≤ 9 indicates absence of depression. The sum score of BDI-S has been shown to be highly reliable (Cronbach's alpha = 0.93)and valid (r = 0.80) [19]. To evaluate sleep quality over a 1-month time interval, the Pittsburgh Sleep Quality Index (PSQI) was applied. With its nineteen questions assessing qualitative and quantitative characteristics of sleep, the instrument displays good reliability (Cronbach’s alpha = 0.77) and validity (r = 0.94). It been translated and cross-culturally adapted for Persian [20]. The Tegner activity score was used to capture the knee-specific functional activity status. It provides a 0–10 point scale with low values representing low activity and high values representing high activity levels. High reliability of the Tegner activity score, which is available in Persian, has been demonstrated (ICC = 0.82) and the validity was r = 0.67 [21]. The CNS Vital Signs battery (CNSVS, Morrisville, North Carolina, USA) was used to assess neurocognitive function. It is a computerized assessment of 22 tasks organized in six modules: stroop test, symbol digit coding, finger tapping, shifting attention, continuous performance as well as visual and verbal memory. The standardized subscale composite scores (verbal memory, visual memory, reaction time, and processing speed) were calculated and used for analysis. Higher scores represent better neurocognitive function. Test–retest reliability has been demonstrated to be high (r = 0.65 to r = 0.88) [22]. Tests were performed in a quiet room to prevent any auditory or visual distraction.

### Data Processing and Statistics

All data were checked for normality using Kolmogorov–Smirnov tests and for variance homogeneity using the Levene’s test. The assumptions for parametric testing were not met. For each biomechanical outcome (TTS, vGRF, COP, joint angles), we computed the mean, minimum and maximum of the six SDL trials. We then performed Mann–Whitney-U tests to detect differences in landing biomechanics and neurocognitive function between the ACLR and CON groups. Cliff’s delta was computed as the effect size and interpreted as follows: < 0.20 = no effect, 0.20 to 0.49 = small effect, 0.50 to 0.79 = moderate effect, and ≥ 0.80 = large effect. Correlations of neurocognitive function and landing biomechanics were examined by means of Spearman’s rank correlation, both for the total sample as well as for the two groups individually. Resulting coefficients were interpreted as negligible (0.00 to 0.20), weak (0.21 to 0.40), moderate (0.41 to 0.60), strong (0.61 to 0.80), and very strong (0.81 to 1.00) according to Akoglu [23]. To identify between-group differences of associations between biomechanics and neurocognitive function, correlation coefficients were transformed to Fisher’s z as proposed by Myers and Sirois [24]. All statistical comparisons were performed with Jamovi (the JAMOVI project). The level of significance was set at *p* < 0.05.

## Results

All individuals completed the experiment without any adverse events.

### Kinematics

Group comparisons revealed no differences for tibial internal rotation (*p* = 0.68 to 0.83) and knee valgus (*p* = 0.92 to 1.0, Table 2). However, relative to CON, the ACLR group exhibited up to 10.4% smaller knee flexion angles (minimum values, ACLR: 50.00° vs. CON: 55.20°; *p* = 0.02).

**Table 2:** Group comparisons of landing kinematics (Mann–Whitney-U test)

Variable	Group	Median	IQR	Test statistic (U)	*p*-value	Effect size (δ)
Max flexion [°]	CON	56.70	53.10–61.10	93.00	0.43	0.17
	ACLR	60.90	54.90–65.00			
Min flexion [°]	CON	55.20	53.00–58.00	59.00	0.02*	0.47
	ACLR	50.00	47.20–53.90			
Mean flexion [°]	CON	53.10	50.10–59.10	91.00	0.38	0.19
	ACLR	56.80	53.60–61.80			
Max valgus [°]	CON	7.43	6.58–8.50	112.00	1.00	0.00
	ACLR	7.52	5.64–8.86			
Min valgus [°]	CON	5.91	4.41–7.88	109.00	0.92	0.03
	ACLR	5.48	4.50–7.05			
Mean valgus [°]	CON	7.11	5.76–7.52	111.00	0.96	0.01
	ACLR	6.67	5.71–7.70			
Max tibial rotation [°]	CON	4.97	4.67–5.69	102.00	0.68	0.09
	ACLR	5.02	3.98–5.62			
Min tibial rotation [°]	CON	2.56	2.43–2.93	104.00	0.74	0.07
	ACLR	2.59	2.24–2.72			
Mean tibial rotation [°]	CON	3.75	3.53–4.50	107.00	0.83	0.04
	ACLR	3.78	3.42–3.88			

All kinematic data were calculated during initial contact. ACLR = Anterior cruciate ligament reconstruction, CON = control, IQR = interquartile range, max = maximum, min = minimum. δ = Cliff’s delta,* significant difference

### Kinetics

TTS values were similar in both groups (*p* = 0.36 to 0.82). Analyses of the other variables revealed systematic differences between CON and ACLR individuals (Table 3). This included a 10.2% higher COP trace lengths (mean, ACLR: 379 mm vs. CON: 344 mm; *p* = 0.04) and a 11.9% (minimum, ACLR: 3.21 N vs. CON: 2.87 N; *p* = 0.01) to 20.3% (mean, ACLR: 3.67 N/kg vs. CON: 3.05 N/kg; *p* = 0.01) higher vGRF in the ACLR group (*p* < 0.05).

**Table 3:** Group comparisons of landing kinetics (Mann–Whitney-U test)

Variable	Group	Median	IQR	Test statistic (U)	*p*-value	Effect size (δ)
Max COP	CON	378.00	325.00–394.00	78.00	0.16	0.30
	ACLR	393.00	338.00–433.00			
Min COP	CON	331.00	294.00–354.00	87.00	0.30	0.22
	ACLR	339.00	321.00–380.00			
Mean COP	CON	344.00	309.00–361.00	64.00	0.04*	0.43
	ACLR	379.00	325.00–397.00			
Max TTS	CON	1.79	1.37–2.11	90.00	0.36	0.20
	ACLR	1.96	1.61–2.27			
Min TTS	CON	1.54	1.24–1.78	105.00	0.77	0.06
	ACLR	1.44	1.18–1.91			
Mean TTS	CON	1.54	1.39–1.66	106.50	0.82	0.05
	ACLR	1.59	1.28–1.87			
Max vGRF	CON	3.59	3.37–4.15	81.00	0.20	0.28
	ACLR	4.04	3.84–4.57			
Min vGRF	CON	2.87	2.46–2.94	55.50	0.01*	0.50
	ACLR	3.21	2.79–3.47			
Mean vGRF	CON	3.05	2.90–3.59	54.00	0.01*	0.52
	ACLR	3.67	3.51–3.99			

All kinetic data (except for TTS) were calculated during initial contact. ACLR = Anterior cruciate ligament reconstruction, CON = contrl, COP = center of pressure, TTS = time to stability, GRF = ground reaction force, v = vertical, max = maximum, min = minimum. δ = Cliff’s delta, * significant difference

### Neurocognitive Function

Visual and (*p* = 0.45) verbal (*p* = 0.85) memory were not different between groups (Table 4). However, the ACLR group displayed lower performance in processing speed (− 13%, ACLR: 82.00 vs. CON: 93.00; *p* = 0.01) and reaction time (− 8%, ACLR: 84.00 vs. CON: 91.00; *p* = 0.02).

**Table 4:** Group comparisons of neurocognitive function (Mann–Whitney-U test)

Variable	Group	Median	IQR	Test statistic (U)	*p*-value	Effect size (δ)
Reaction time	CON	91.00	86.00–102.00	59.00	0.02*	0.47
	ACLR	84.00	62.00–91.00			
Processing speed	CON	93.00	81.00–96.00	53.00	0.01*	0.52
	ACLR	82.00	71.00–82.00			
Visual memory	CON	98.00	88.00–108.00	94.00	0.45	0.16
	ACLR	104.00	81.00–113.00			
Verbal memory	CON	105.00	102.00–109.00	107.00	0.85	0.04
	ACLR	105.00	101.00–109.00			

Higher scores indicate higher neurocognitive performance. ACLR = Anterior cruciate ligament reconstruction, CON = control, IQR = interquartile range, δ = Cliff’s delta, *significant difference

### Associations Between Landing Kinetics and Neurocognitive Function

All correlation coefficients are displayed in full detail in Table 5. Reaction time and visual memory were not associated with landing kinetics (*p* > 0.05). However, significant associations were found for verbal memory and processing speed.

**Table 5:** Correlations between Neurocognitive Function and Landing Kinetics

Variables			Correlation coefficients			Δ of group correlations: *p* (z-value)
			Full dataset	CON group	ACLR group	
Verbal memory	vGRF	Max	− 0.45*	− 0.62*	− 0.38	0.21 (0.79)
		Min	− 0.34	− 0.75*	− 0.19	0.02* (1.91)
		Mean	− 0.44	− 0.58*	− 0.40	0.27 (0.58)
	TTS	Max	− 0.16	− 0.07	− 0.36	0.22 (0.75)
		Min	− 0.27	− 0.18	− 0.34	0.33 (0.42)
		Mean	− 0.08	− 0.16	− 0.04	0.38 (0.29)
	COP	Max	0.26	− 0.14	− 0.33	0.31 (0.49)
		Min	0.32	− 0.16	− 0.50*	0.17 (0.95)
		Mean	0.20	0.11	− 0.40	0.09 (1.31)
Visual memory	vGRF	Max	− 0.17	− 0.02	− 0.41	0.15 (1.01)
		Min	− 0.24	− 0.16	− 0.40	0.26 (0.64)
		Mean	− 0.09	− 0.09	− 0.36	0.24 (0.70)
	TTS	Max	− 0.03	− 0.11	− 0.04	0.43 (0.17)
		Min	− 0.05	− 0.01	− 0.05	0.46 (0.09)
		Mean	− 0.06	− 0.14	− 0.07	0.43 (0.17)
	COP	Max	0.01	0.00	− 0.09	0.41 (0.22)
		Min	− 0.12	0.30	− 0.20	0.10 (1.25)
		Mean	− 0.30	0.23	− 0.07	0.22 (0.75)
Processing speed	vGRF	Max	− 0.24	− 0.36	− 0.40	0.37 (0.15)
		Min	− 0.59*	− 0.52*	− 0.47*	0.43 (0.16)
		Mean	− 0.64*	− 0.65*	− 0.46*	0.24 (0.68)
	TTS	Max	− 0.30	− 0.11	− 0.31	0.30 (0.51)
		Min	− 0.24	− 0.13	− 0.42	0.21 (0.77)
		Mean	− 0.18	− 0.14	− 0.36	0.28 (0.57)
	COP	Max	− 0.23	− 0.16	− 0.35	0.30 (0.50)
		Min	− 0.15	− 0.05	− 0.25	0.30 (0.50)
		Mean	− 0.17	− 0.05	− 0.28	0.28 (0.58)
Reaction time	vGRF	Max	− 0.05	− 0.04	− 0.03	0.49 (0.02)
		Min	− 0.21	− 0.26	− 0.14	0.38 (0.30)
		Mean	− 0.12	− 0.08	− 0.07	0.49 (0.02)
	TTS	Max	− 0.16	− 0.06	− 0.11	0.45 (0.12)
		Min	− 0.36	− 0.34	− 0.17	0.12 (1.16)
		Mean	− 0.19	− 0.35	− 0.16	0.30 (0.50)
	COP	Max	− 0.05	0.31	0.16	0.34 (0.39)
		Min	− 0.01	0.10	0.21	0.39 (0.27)
		Mean	− 0.11	0.14	0.17	0.47 (0.07)

Table shows Spearman’s Rho correlation coefficients. Significant associations are marked bold and with an asterisk. All kinematic data are expressed as [°]. ACLR = anterior cruciate ligament reconstruction, CON = control, TTS = time to stabilisation, vGRF = vertical ground reaction force, COP = center of pressure, max = maximum, min = minimum

With regard to verbal memory, a higher capacity correlated with a smaller length of the COP trace in the ACLR group only (r =  − 0.50, *p* < 0.05). In contrast, higher verbal memory scores were associated with lower GRF values in the control group only (r =  − 0.58 to − 0.75, *p* < 0.05) and the group comparison of the coefficients was significant (*p* = 0.02, z = 1.91). With regard to processing speed, a better test performance correlated with lower vGRF values in both groups (r = − 0.46 to − 0.65, *p* < 0.05) and no between-group differences were found (*p* > 0.05).

### Associations Between Landing Kinematics and Neurocognitive Function

All correlation coefficients are displayed in full detail in Table 6. Significant associations were detected for all neurocognitive functions.

**Table 6:** Correlations between neurocognitive function and landing kinematics

Variables			Correlation coefficients			Δ of group correlations: *p* (z-value)
			Full dataset	CON group	ACLR group	
Verbal memory	FLX	Max	0.01	0.15	0.07	0.42 (0.19)
		Min	0.09	0.26	0.03	0.28 (0.57)
		Mean	0.03	0.18	0.00	0.32 (0.44)
	VLG	Max	− 0.33	− 0.22	− 0.42	0.19 (0.86)
		Min	− 0.00	− 0.05	− 0.37	0.20 (0.82)
		Mean	− 0.20	− 0.00	− 0.57*	0.05 (1.58)*
	TIBIA ROT	Max	0.02	− 0.13	− 0.12	0.49 (0.02)
		Min	− 0.15	− 0.28	0.16	0.13 (1.10)
		Mean	0.18	− 0.12	0.09	0.30 (0.52)
Visual memory	FLX	Max	0.33	0.04	0.51*	0.10 (1.28)
		Min	0.10	0.28	0.25	0.46 (0.07)
		Mean	0.35	0.13	0.53*	0.04 (1.12)*
	VLG	Max	0.00	− 0.20	− 0.28	0.41 (0.20)
		Min	0.00	− 0.18	− 0.06	0.38 (0.29)
		Mean	− 0.01	− 0.05	− 0.17	0.38 (0.29)
	TIBIAL ROT	Max	− 0.55*	− 0.49*	− 0.58*	0.37 (0.31)
		Min	− 0.35*	− 0.27	− 0.40	0.36 (0.36)
		Mean	− 0.08	− 0.33	0.10	0.13 (1.09)
Processing speed	FLX	Max	0.09	0.14	0.25	0.39 (0.28)
		Min	0.64*	0.41	0.63*	0.22 (0.74)
		Mean	0.19	0.26	0.31	0.44 (0.13)
	VLG	Max	− 0.22	− 0.25	− 0.15	0.39 (0.25)
		Min	− 0.23	− 0.19	− 0.12	0.43 (0.17)
		Mean	− 0.07	− 0.01	− 0.09	0.42 (0.19)
	TIBIAL ROT	Max	− 0.01	− 0.01	− 0.11	0.40 (0.24)
		Min	− 0.02	− 0.38	− 0.12	0.24 (0.68)
		Mean	− 0.27	− 0.37	− 0.33	0.45 (0.11)
Reaction time	FLX	Max	0.31	0.50*	0.30	0.27 (0.58)
		Min	0.62*	0.44*	0.46*	0.47 (0.06)
		Mean	0.32	0.52*	0.25	0.21 (0.78)
	VLG	Max	− 0.13	− 0.15	− 0.23	0.41 (0.20)
		Min	− 0.35	− 0.34	− 0.26	0.18 (0.89)
		Mean	− 0.29	− 0.31	− 0.26	0.44 (0.13)
	TIBIAL ROT	Max	− 0.20	− 0.36	− 0.09	0.24 (0.70)
		Min	− 0.40	− 0.47	− 0.42	0.43 (0.15)
		Mean	− 0.12	− 0.03	− 0.29	0.25 (0.65)

Table shows Spearman’s Rho correlation coefficients. Significant associations are marked bold and with an asterisk. All kinematic data are expressed as [°]. ACLR = anterior cruciate ligament reconstruction, CON = control, FLX = Flexion, VLG = Valgus, ROT = Rotation, max = maximum, min = minimum

In the ACLR group only, better verbal memory performance correlated with lower knee valgus angles (r = -0.57, *p* < 0.05) and the group comparison of the coefficients was significant (*p* = 0.05, z = 0.158). Also, higher visual memory scores correlated with higher knee flexion angles in the ACLR group only (r = 0.51, *p* < 0.05) although the group comparison marginally failed significance (*p* = 0.10). In both groups, better visual memory was linked to lower tibial rotation angles and consequently, there was no difference in the correlation coefficients. A lower processing speed correlated with smaller knee flexion angles in the ACLR group only (r = 0.63, *p* < 0.05) but the group comparison was not significant (*p* = 0.22). Finally, shorter reaction times correlated with more knee flexion in ACLR and CON (r = 0.44 to 52, *p* < 0.05), but there was no between-group difference.

## Discussion

So far, the role of time-constrained decision-making during athletic movement had mostly been investigated in healthy individuals [10]. Even in this population, unplanned motor actions such as spontaneous changes of direction are associated with unfavorable knee mechanics [25]. Our study adds that ACLR athletes, compared to non-injured controls, display further impairments in kinetics (e.g., increased GRF) and kinematics (e.g., decreased knee flexion angle) which may explain their higher risk of re-injury [26]. Of note, landing biomechanics (i.e., knee kinematics) correlated strongly with markers of neurocognition, meaning that a low memory capacity and a low processing speed are linked to impaired movement safety. Associations most often included knee flexion and ACLR participants displayed both smaller joint angles and lower cognitive test performance. This may mean that higher knee flexion angles, which are assumed to lower the stress on the ACL [27], can only be achieved if sensory perception and stimulus processing are sufficiently fast.

Although there had been a general paucity of trials on decision-making in injured athletes, Giesche et al. [26] examined unplanned unilateral jump landings following surgical treatment of ACL rupture. The authors focused on cortical aspects of motor planning and some biomechanical variables, which, however, did not include knee joint kinematics. They reported no differences between ACLR participants and controls in terms of TTS, GRF, and COP. The reasons for the contrast of these findings towards our trial could relate to the selection of the visual cue and the examined sample. While Giesche and colleagues [26] applied a rather simple stimulus (arrow) with two choices, our participants had to select from three options, recognizing colors. Also, we exclusively recruited elite soccer players instead of recreational athletes from both open and close skill sports.

The observed differences in knee mechanics between ACLR and CON athletes as well as the observed associations with neurocognitive function may have clinical implications. A variety of motor deficits linked to ACL injury, e.g., decrements in strength, power, balance, range of motion, or limb symmetry, can be eliminated almost entirely during the return to play process following ACL rupture [28]. However, although we cannot assume cause-effect relationships, our findings indicate that potential dysfunctions in neurocognitive decision-making may not be restored by conventional rehabilitation paradigms. This would fit with data from fMRI studies showing substantial cortical reorganization in ACLR patients [6]. If lasting neurocognitive deficits would in fact explain the high probability of re-ruptures [3], this could alter exercise habits in the prevention of ACL tears. Interestingly, currently available programs such as the Prevent Injury and Enhance Performance (PEP) program [29] do not include any significant cognitive/decision-making components. Such, however, could be paramount when aiming to regain pre-injury function. Practitioners may hence consider adding unplanned exercise drills in preventive and therapeutic programs and, in addition, training neurocognitive functions of their athletes. With regard to improvements in generic cognitive skills, researchers have questioned the far transfer to sports performance [30, 31]. Notwithstanding, a study using a 6-week intervention with purely computerized exercise indeed improved lower leg choice-reaction performance, which could be of use in landing situations [32].

While our study yields interesting findings potentially pointing towards a role of neurocognitive function in ACL injury, the specific neural factors underlying biomechanical alterations in ACLR athletes remain speculative. Previous studies suggest that cortical processing of visual input may play a key role during decision-making. Grooms et al. [33] used strobe classes to obstruct vision in ACLR and healthy individuals performing drop landings. Trials with glasses produced higher GRF and knee flexion excursions than landings with unrestricted sight [33]. In a similar study, Santello et al. [34] compared drop landings with open and closed eyes. Without vision, GRF were 10% higher and knee joint rotation angles lower [34]. Chu et al. [35] studied the effect of vision on the safety of double-leg landings in air assault soldiers. Blindfolded landings increased hip abduction at initial contact and maximal GRF while reducing maximal knee flexion [35]. Finally, Brazalovich et al. [36] found that wearing a head-mounted virtual reality display during drop landing decreases knee flexion, knee abduction as well as scores of the landing error scoring system when compared to normal vision and no vision. It has been assumed that reduced afferent input and altered central processing following ACL injury [37] place excessive demands on the visual cortex, which, in fact, exhibits increased activity in ACL patients [38]. Athletes with a history of injury, furthermore, have a reduced connectivity between the primary sensory cortex and the cerebellum [39] as well as between the left somatosensory cortex and a variety of motor regions including the supplementary motor area, the pre-motor cortex, and the primary motor cortex [39]. All these alterations, acting in concert, could increase the total time needed for perception–action coupling and hence reduce the time available for dynamic joint stabilization. Our findings are basically in line with this theory as most associations between landing biomechanics and neurocognitive function were unfavorable for the ACLR but not the control group.

Some shortcomings need to be discussed. First, due to the exploratory nature of our trial as well as its complex design and outcomes, the sample size (n = 30) and the resulting statistical power were comparatively small. Interestingly, we found a variety of differences between both groups. However, additional confirmatory investigations corroborating and extending our results would be welcome to further strengthen our conclusions. A second aspect relates to the experimental task. Although we asked our participants to respond to the visual cue after take-off, we cannot exclude completely the possibility that some individuals may have guessed the landing side or followed their predefined motor plans, regardless of the presented stimulus. However, this rather generic limitation applies to all trials including decision-making. Third, our study did not include an unplanned condition without a reactive stimulus. It has been argued that athletes with ACLR would exhibit differences in jump-landing biomechanics, which, inter alia, include reduced knee flexion angles. Available systematic reviews with meta-analysis, however, provide conflicting results. While Johnston et al. [40] indeed reported lower knee flexion values in individuals with ACLR, Lepley et al. [41] found no differences between controls and ACLR athletes in this parameter. Despite the lack of conclusive evidence, future studies should involve both, unplanned and planned landing tasks in order to further delineate the role of reactive decision-making on knee mechanics. Finally, we were unable to document the graft type used for ACL surgery. Although the meta-analyses of Johnston et al. [34] and Lepley and Kuenze [35] concluded that there is insufficient data on the impact of the graft material on jump landing biomechanics, it would have been of interest to look into this variable.

Several aspects call for further research. Besides other variables, we found ACLR athletes to display higher minimal GRF (averaged over the six jumps) during landing. To the best of our knowledge, this parameter has not been extensively studied with regard to its potential value in injury prediction. It would hence be of interest to include it in future trials. Furthermore, upcoming research may combine the applied biomechanical markers and set-ups with additional outcomes such as electromyography or fMRI investigations revealing muscle activity and cortical activation patterns. Furthermore, as our and almost all previous studies had a cross-sectional design, longitudinal studies and prospective trials are warranted in order to further elucidate potential causal relationships between neurocognitive athletic decision-making and the occurrence of lower limb injury. In case of clinically relevant associations, randomized, controlled trials should be performed testing the effectiveness of training and/or warm-up interventions aiming to improve movement-related cognitive skills in both healthy persons and athletes with a history of injury.

## Conclusions

Individuals with ACLR exhibit kinematic and kinetic knee impairments during neurocognitively challenged drop landings. These biomechanical deficits (i.e. knee kinematics) correlate with lower cognitive functions such as processing speed and memory capacity and may increase injury risk. Coaches and therapists should hence consider the use of specifically tailored testing and training paradigms seeking to improve time-constrained decision-making.

## Data Availability

The datasets generated during and/or analyzed during the current study are available from the corresponding author on reasonable request.

## Abbreviations

ACL: Anterior cruciate ligament
ACLR: Anterior cruciate ligament reconstruction
CON: Control group
GRF: Ground reaction force
TTS: Time to stabilization
COP: Center of pressure
PEP: Prevent injury and enhance performance
